# Quinoa Ameliorates High‐Fat Diet‐Induced Obesity in Female Mice by Regulating Gut Microbiota and Adipogenesis

**DOI:** 10.1002/fsn3.71250

**Published:** 2025-11-25

**Authors:** Yingqi Liu, Qiwen Pan, Xiaohua Bao, Yingxin Zhang, Xianjun Liu, Yue Liu, Yujie Liu, Yingxin Zhang, Qirui Xie, Zhiyong Liang, Fengjie Sun, Jing Li, Hao Li, Zhandong Li

**Affiliations:** ^1^ College of Biological and Food Engineering Jilin Engineering Normal University Changchun China; ^2^ Department of Gastroenterology Chinese People's Liberation Army 964 Hospital Changchun China; ^3^ Faculty of Mathematics University of Waterloo Waterloo Canada; ^4^ Qingdao Haoda Marine Biotechnology Co., Ltd Qingdao China; ^5^ Department of Biological Sciences, School of Science and Technology Georgia Gwinnett College Lawrenceville Georgia USA; ^6^ School of Computer Science Baicheng Normal University Baicheng China

**Keywords:** 16S rRNA, gut microbiota, high‐fat diet, ion channel, quinoa, transcriptomics

## Abstract

The female obesity epidemic remains a major global public health challenge, with limited effective treatments available. Daily dietary management serves as an effective strategy for obesity prevention. As a promising functional food, quinoa has been demonstrated to possess anti‐obesity properties. However, the mechanisms of quinoa on high‐fat diet (HFD)‐induced female obesity and its role in regulating adipose tissue remain unclear. In the present study, the effects of different dosages of quinoa on obesity and its underlying mechanisms were investigated in an HFD‐induced mouse model of obesity. Six‐week‐old female C57BL/6J mice were fed an HFD to induce obesity, followed by an 8‐week intervention with low dosage of quinoa (LQ), medium dosage of quinoa (MQ), and high dosage of quinoa (HQ), respectively. Then, 16S rRNA gene sequencing was performed to investigate gut microbial community and transcriptome analysis was conducted to characterize differentially expressed genes (DEGs) in gonadal white adipose tissue (gWAT) in mice. The HQ group significantly attenuated body weight in HFD‐fed mice. The 16S rRNA gene sequencing analysis revealed that the three intervention groups exhibited distinct bacterial community profiles, and the bacterial taxa significantly enriched in response to quinoa intake were identified. Notably, the HQ group modulated gut microbiota composition, demonstrating significant regulatory effects on the populations of Bacteroidota, Bacteroidia, Bacteroidales, and Muribaculaceae, while concurrently reducing the Firmicutes‐to‐Bacteroidetes (F/B) ratio. Furthermore, transcriptomic analysis revealed that HQ treatment modulated HFD‐induced DEGs associated with pathways of “transmembrane transporter complex,” “ion gated channel activity,” and “calcium ion binding” in gWAT. These findings suggest that quinoa alleviates HFD‐induced obesity by regulating community structure of gut microbiota and the ion channel in gWAT, providing a strong theoretical foundation for the development of quinoa products for the treatment of female obesity.

## Graphical abstract

Quinoa intervention counteracts high‐fat diet (HFD)‐induced obesity in C57BL/6 female mice and elicits a concomitant increase in the relative abundance of Bacteroidota, Bacteroidia, Bacteroidales, and Muribaculaceae. Treatment with quinoa in HFD‐fed female mice results in reduced expression of genes involved in “ion channel” in gonadal white adipose tissue (gWAT), which is associated with decreased adiposity. These findings demonstrate that quinoa mitigates HFD‐induced metabolic disruption by concurrently ameliorating gut microbiota dysbiosis and gWAT dysfunction.

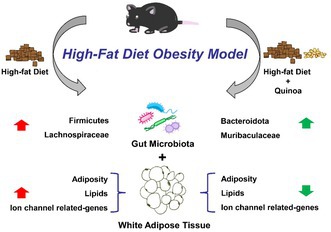

## 1 Introduction

Obesity triggers a cascade of systemic metabolic alterations, including insulin resistance, dyslipidemia, hypercholesterolemia, and chronic low‐grade inflammation (Deng et al. 2016). The rising prevalence of obesity is fueled by imbalances in lipid catabolism (Axelrod et al. 2023; Chen et al. 2020; Sakers et al. 2022). During its pathogenesis, WAT upregulates genes linked to obesity, which correlate with compromised lipid catabolism (Greenhill 2024; Kirstein et al. 2021; Kusminski et al. 2016; Nigro et al. 2023; Sun et al. 2011). Current preventive strategies—including diet, exercise, pharmacotherapy, and bariatric surgery—are now being complemented by a growing research focus on functional foods. These nutritionally tailored interventions represent a promising, safer, and more sustainable avenue for combating obesity and enhancing public health.

The gut harbors a vast and diverse ecosystem of microorganisms that are indispensable for host development and physiological homeostasis (Kawano et al. 2022; Loo et al. 2017; Neto et al. 2020). A substantial body of evidence now underscores the pivotal role of the gut microbiome in body weight regulation, with specific microbial communities able to orchestrate either beneficial or detrimental metabolic outcomes (Cho et al. 2012; Reijnders et al. 2016; Turnbaugh et al. 2008; Turnbaugh et al. 2006; Zou et al. 2023). For example, a rise in the Firmicutes/Bacteroidetes (F/B) ratio and specific shifts in microbiota composition are hallmarks of obesity (Turnbaugh et al. 2006). Prebiotics, which comprise non‐digestible fermentable carbohydrates and fibers, ameliorate these effects by selectively enriching beneficial gut microbiota, leading to reduced adiposity and systemic inflammation (Everard et al. 2011; Nakamura and Omaye 2012). Nevertheless, the precise pathway by which prebiotics reshape gut microbial communities and inhibit fat accumulation, thereby counteracting obesity, has not been fully elucidated.

Quinoa ( *Chenopodium quinoa* Willd.) is a nutrient‐dense pseudocereal characterized by a complete essential amino acid profile, high‐quality lipids, substantial dietary fiber, and a diverse array of bioactive secondary metabolites such as steroids, flavonoids, and triterpenoid saponins (Abugoch James 2009). These active compounds endow quinoa with multiple health benefits, including antibacterial, blood sugar‐lowering, anti‐inflammatory, and immunomodulatory effects (Ren et al. 2023; Simnadis et al. 2015; Tan et al. 2021). Previous research suggests that quinoa may possess prebiotic properties, mediating the growth of commensal gut microbiota and enhancing short‐chain fatty acid (SCFA) biosynthesis (Liu et al. 2018). As a valuable functional and medicinal food, quinoa has demonstrated potential benefits in managing obesity and related endocrine diseases (Sharma et al. 2025). Although the efficacy of quinoa against obesity has been extensively investigated, its underlying mechanisms remain unclear.

In the present study, we elucidate the modulatory effects of quinoa on HFD induced obesity through analyze the gut microbiome alteration, thereby gaining insights into the functional roles of quinoa in obesity amelioration. We explored the dose‐dependent effects of quinoa consumption on gut microbiota composition in HFD‐fed mice and the transcriptome analysis of gWAT were also investigated. This study establishes a theoretical foundation for further identifying the targets of quinoa to ameliorates obesity, which is critical for promoting the comprehensive application of quinoa.

## 2 Methods

### 2.1 Animals and Diets

All animal procedures were performed in accordance with the Institutional Animal Care and Use Committee (IACUC) guidelines under an approved protocol by the IACUC of Jilin Engineering Normal University (Approval number 2024SY10502). Female C57BL/6J mice (6 weeks old) of specific pathogen‐free (SPF) grade (with an average body weight of 14 ± 2 g) were purchased from Liaoning Changsheng Biotechnology Co. Ltd. (Benxi, Liaoning, China) and maintained in separate cages (5 mice per cage) under controlled environmental conditions (22°C ± 2°C and 40%–50% humidity) with a 12‐h/12‐h light/dark photoperiod cycle. Mice were acclimated for 1 week with free access to water and a standard commercial diet, which was purchased from Xietong Bioscience Co. Ltd. (Nanjing, Jiangsu, China).

### 2.2 Obesity Induction and Intervention Procedure

The quinoa was obtained from Huaqiangu Stone Flour Processing Co. Ltd. (Tongyu, Jilin, China). Following the acclimatization period, mice were randomly assigned to receive either a standard diet or a high‐fat diet (HFD; 60% kcal derived from fat) for 8 weeks (Table S1). The standard diet (XTCON50J) and HF diet (XTHF60) were purchased from Xietong Bioscience Co. Ltd. (Nanjing, Jiangsu, China). Then, mice fed the HFD that exhibited ≥ 20% higher body weight compared to standard diet‐fed mice were classified as obese models. Mice with established obesity were randomly assigned to the HFD‐induced obesity (HFD) group, “HFD + low dosage of quinoa” (LQ) group, “HFD + medium dosage of quinoa” (MQ) group, and “HFD + high dosage of quinoa” (HQ) group (each group contained 5 mice). The control group was maintained on a standard diet, while the HFD group remained on HFD. The LQ group was fed quinoa (daily dose of 1 g was administered to each mouse) maintained with HFD, the MQ group was fed quinoa (daily dose of 1.5 g was administered to each mouse) maintained with HFD, and the HQ group was fed quinoa (daily dose of 2 g was administered to each mouse) maintained with HFD. Following oral administration of 1, 1.5, or 2 g quinoa to each mouse, animals were subsequently allowed *ad libitum* access to the high‐fat diet. During the 8‐week intervention, body weight and food intake were measured at the end of each week. After the experimental period, mice were sacrificed under anesthesia. Blood samples were collected by enucleation (eyeball removal) for further analysis. The gWAT was immediately collected, weighed, and stored at −80°C for further analysis.

### 2.3 Serum Biochemical Analysis

Eyeball blood was collected in vacuum tubes, blood samples were allowed to clot at room temperature for 2 h, and centrifuged to obtain serum supernatant. Following centrifugation, the supernatant was collected for quantitative analysis of serum lipid profiles, including total cholesterol (TC), triglycerides (TG), low‐density lipoprotein cholesterol (LDL‐C), and high‐density lipoprotein cholesterol (HDL‐C) concentrations using a microplate spectrophotometer (BioSino Bio‐technology and Science Inc., Beijing, China) following the instructions of the manufacturer. Levels of aspartate transaminase (AST), and alanine aminotransferase (ALT) were measured using commercial kits (Jiancheng Co., Nanjing, Jiangsu, China).

### 2.4 Histopathological Analysis

Fixed small intestine tissues were processed through standard histological protocols, embedded in paraffin, and sectioned for hematoxylin–eosin (H&E) staining. Paraffin‐embedded tissue sections (3–5 μm thick) were examined and evaluated using light microscopy (Olympus Soft Imaging Solutions GmbH, Münster, Germany). Adipocyte area was quantified using ImageJ software. To ensure measurement reliability, adipocytes with an area less than 50 μm^2^ and those located along the tissue edges were excluded from the analysis.

### 2.5 Villi Length Measurement

Morphometric analysis of villus length was performed using ImageJ software. Only villi that were intact and oriented along their full length were measured from the crypt‐villus junction to the tip. Any villi that were broken, cross‐cut, or degraded were excluded from the analysis. For each mouse, 10 villi were measured, and the mean villus length was calculated and reported.

### 2.6 DNA Extraction and 16S rRNA Gene V34 Region Sequencing

Fecal samples from the mice were collected and stored at −80°C for further analysis. Bacterial populations in feces were determined using high‐throughput gene sequencing of V34 variable regions of the bacterial 16S rRNA gene. The 16S rRNA gene was PCR‐amplified with V34 region‐specific primers (forward: 5′ CCTAYGGGRBGCASCAG‐3′; reverse: 5′ GGACTACNNGGGTATCTAAT‐3′). Fecal genomic DNA of feces was extracted using the DNA extraction kit (TIANGEN, Beijing, China) and used as templates to amplify the V34 region of the 16S rRNA gene. Sequencing libraries were constructed with dual‐index adapters, then quantified using Qubit fluorometry and qPCR, and assessed for size distribution by bioanalyzer analysis. Based on quality control metrics, DNA libraries were normalized to equimolar concentrations, pooled, and sequenced on an Illumina platform (Illumina, San Diego, CA, USA) to achieve the desired sequencing depth.

### 2.7 Microbiome Bioinformatics Analysis

Paired‐end reads of 16S rRNA gene sequencing were merged using FLASH (v1.2.11) (Magoč and Salzberg 2011). The resulting concatenated sequences were designated as raw tags. Subsequent quality filtering was performed using fastp (v0.23.1) (Bokulich et al. 2013) to generate high‐quality clean tags. Sequence tags were aligned against the SILVA reference database (16S; v138) for chimera detection using VSEARCH (v2.16.0). Chimera‐filtered effective tags were subsequently processed through the QIIME2 pipeline (v2021.4), with denoising performed via the default DADA2 module to generate amplicon sequence variants (ASVs). Taxonomic annotation of ASVs was then conducted using the classification algorithms of QIIME2. 16S rRNA gene sequencing analysis was taxonomically classified using the SILVA reference database.

All downstream α‐diversity and β‐diversity analyses were performed using the normalized data. For taxonomic composition visualization, the top 20 most relatively abundant taxa at each rank (phylum to genus) were selected to generate relative abundance histograms using the SVG module of Perl. Venn diagrams were constructed using both R (VennDiagram package) and Perl (SVG module) for comparative purposes. Gut microbiota α‐diversity metrics (e.g., Shannon index and observed ASVs) were computed through the core diversity metrics pipeline of QIIME2, containing the Chao1 estimator (http://scikit‐bio.org/docs/latest/generated/skbio.diversity.alpha.chao1.html), the Dominance index (http://scikit‐bio.org/docs/latest/generated/skbio.diversity.alpha.dominance.html), the Shannon index (http://scikit‐bio.org/docs/latest/generated/skbio.diversity.alpha.shannon.html), the Simpson index (http://scikit‐bio.org/docs/latest/generated/skbio.diversity.alpha.simpson.html), and Pielou's evenness index (http://scikit‐bio.org/docs/latest/generated/skbio.diversity.alpha.pielou_e.html).

To assess microbial community complexity and inter‐sample differences, β‐diversity was analyzed using both weighted and unweighted UniFrac distances in QIIME2. Principal coordinate analysis (PCoA) was performed to reduce these multidimensional distance matrices into orthogonal axes, where sequential coordinates captured decreasing proportions of variance (PC1: maximum variation; PC2: secondary variation, etc.). The resulting PCoA plots were visualized using R (v4.0.3) in the ade4 package for coordinate transformation and ggplot2 for graphical representation. Non‐metric multidimensional scaling (NMDS) was employed for dimensionality reduction. While similar to PCoA in utilizing distance matrices, NMDS specifically preserved the rank‐order relationships among samples rather than absolute numerical distances. This ordination approach was implemented in R (v4.0.3) using the ade4 package for computation and ggplot2 for visualization, with stress values calculated to evaluate representation quality.

The linear discriminant analysis effect size (LEfSe) algorithm was employed to identify differentially abundant microbial taxa between groups, using an LDA score threshold of > 4.0 and a significance level of *p* < 0.05.

### 2.8 Transcriptome Sequencing Analysis

Total RNA was extracted from fresh tissues of gWAT (80 mg) collected from each group of mice and processed by poly (A) selection using magnetic beads, followed by fragmentation using 5× First Strand Synthesis Buffer (Thermo Fisher, Shanghai, China) with divalent cations at elevated temperature. First‐strand cDNA synthesis was performed using random hexamer primers and M‐MuLV Reverse Transcriptase (RNase H^−^), followed by second‐strand cDNA generation with DNA Polymerase I and RNase H of the RNA Library Prep kit (NEB, Ipswich, USA). PCR‐amplified libraries were purified with AMPure XP beads and assessed for quality using an Agilent Bioanalyzer 2100 (Agilent Technologies, Shanghai, China).

The reference genomic sequence and gene annotation files were retrieved from the genome portal (http://ftp.ensembl.org/pub/release‐94/gtf/homo_sapiens/Homo_sapiens.GRCh38.94.gtf.gz). Genomic alignment indices were generated using HISAT2 (version 2.0.5), followed by mapping of the processed paired‐end sequencing reads to the reference assembly through the HISAT2 aligner using default parameters. Transcriptomic comparison among experimental groups was conducted using the DESeq2 package (v1.20.0) in R. DESeq2 employed a negative binomial distribution model to statistically assess the differences in digital gene expression data. Adjusted *p*‐values were established using the Benjamini‐Hochberg false discovery rate (FDR) correction. Differentially expressed genes (DEGS) were identified based on *p* < 0.05 by DESeq2. Raw sequencing data were deposited to the National Center for Biotechnology Information (NCBI) Sequence Read Archive (SRA) database (https://www.ncbi.nlm.nih.gov/sra/) under the accession number PRJNA1279820.

Functional enrichment analyses of DEGs based on Gene Ontology (GO) and Kyoto Encyclopedia of Genes and Genomes (KEGG) were performed using the clusterProfiler R package. Both GO terms and KEGG pathways were analyzed with gene length bias correction, and statistically significant terms/pathways were determined based on adjusted *p* < 0.05.

### 2.9 Real‐Time qPCR

Quantitative real‐time PCR (qPCR) was conducted using SYBR Green Realtime qPCR Master Mix (TOYOBO, Osaka, Japan) on a LightCycler 480 system (Roche, Boston, MA, USA). β‐actin served as the internal control gene, with relative gene expression quantified using the comparative 2^−ΔΔCt^ method to calculate the relative expression of genes. All qPCR primers were commercially synthesized by ComateBio Co. Ltd. (Changchun, Jilin, China) (Table S2).

### 2.10 Statistical Analysis

All quantitative data were expressed as mean ± standard deviation (SD) and were statistically analyzed using GraphPad Prism software (San Diego, CA, USA). Statistical comparisons between two groups were performed using Student's *t*‐tests. For multi‐group analyses, one‐way ANOVA with Tukey's multiple comparison test was applied to assess statistical significance across the five experimental groups based on *p* < 0.05.

## 3 Results

### 3.1 Quinoa Diet Reduced Obesity in HFD Mice

**FIGURE 1:**
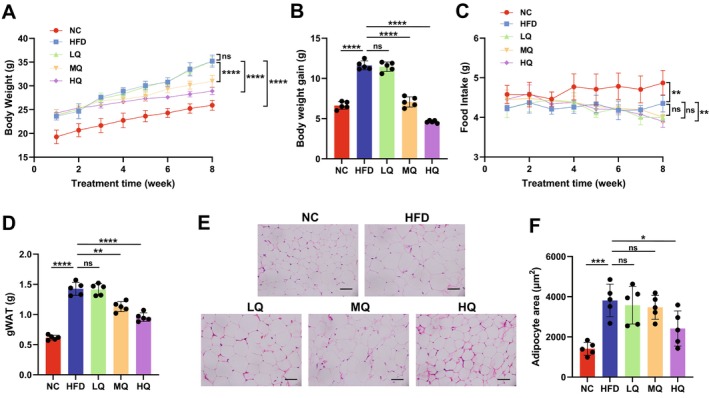
Quinoa reduces the obesity level in HFD mice. (A) Growth of body weight. (B) Changes in body weight growth. (C) Food intake. (D) gWAT depot weights. (E) Representative images of H&E staining of gWAT sections. (F) Quantification of gWAT adipocyte area. Data are presented as the mean ± SD. Statistical significance is determined by *p* < 0.0001 (****), *p* < 0.001 (***), *p* < 0.01 (**), and *p* < 0.05 (*), respectively; ns, not significant. Scale bar = 50 μm.

During the 8‐week intervention, HFD‐fed mice exhibited accelerated weight gain compared to controls, and both MQ and HQ interventions significantly suppressed HFD‐induced weight gain (Figure 1A,B). In contrast to the HQ intervention, the LQ intervention failed to attenuate HFD‐induced weight gain (Figure 1A,B). Compared with the control group, the food intake in the HFD group decreased from week 4 to week 8 (Figure 1C). The HQ groups showed a significant alteration in food intake compared to the HFD group (Figure 1C). Moreover, the weight of the gWAT from HFD mice appeared apparently higher than controls, and a lower gWAT weight was observed in HQ‐fed mice compared to the HFD group (Figure 1D). Hematoxylin and eosin (H&E) staining of gWAT sections revealed that adipocytes in HFD‐fed mice were significantly larger, while the HQ intervention markedly attenuated this HFD‐induced adipocyte hypertrophy (Figure 1E,F). Previous studies have indicated that feeding HFD to mice resulted in a shortening of small intestinal villus length (Beyaz et al. 2016). After the HQ intervention, the intestinal villus length was significantly rescued compared to the HFD group (Figure S1). These findings indicated that HQ interventions attenuated HFD‐induced obesity in mice, with the HQ group showing superior efficacy.

**FIGURE 2:**
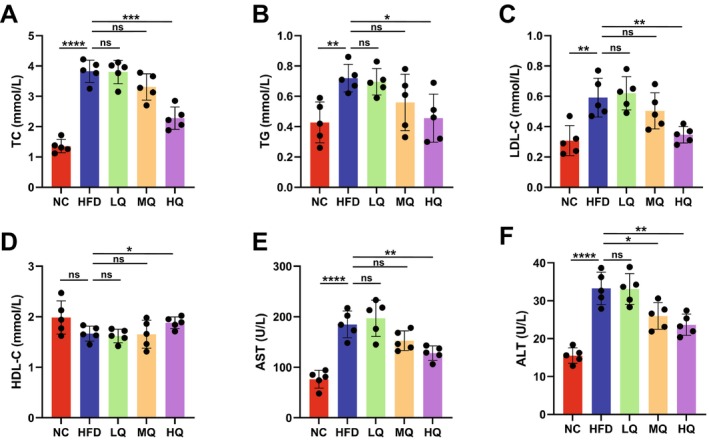
Levels of six serum biomarkers in five groups of mice under the treatments of HFD and quinoa. (A) Total cholesterol (TC). (B) Serum triglyceride (TG). (C) Low‐density lipoprotein cholesterol (LDL‐C). (D) High‐density lipoprotein cholesterol (HDL‐C). (E) Aspartate aminotransferase (AST). (F) Alanine aminotransferase (ALT). Data are presented as the mean ± SD. Statistical significance is determined by *p* < 0.0001 (****), *p* < 0.001 (***), *p* < 0.01 (**), and *p* < 0.05 (*), respectively; ns, not significant.

Following the 8‐week intervention period, blood samples were collected for biomarker quantification. Compared to controls, the HFD group revealed markedly upregulated levels of TC, TG, LDL‐C, AST, and ALT (Figure 2), while the HQ group exhibited significantly lower levels of TC, TG, LDL‐C, AST, and ALT than the HFD group (Figure 2). Taken together, these findings indicated that HFD effectively recapitulated obesity and metabolic dysfunctions, while HQ supplementation demonstrated significant potential to modulate HFD‐driven adipogenesis and its pathological consequences.

### 3.2 Quinoa Diet Modulated the Gut Microbiota of HFD‐Induced Obese Mice

**FIGURE 3:**
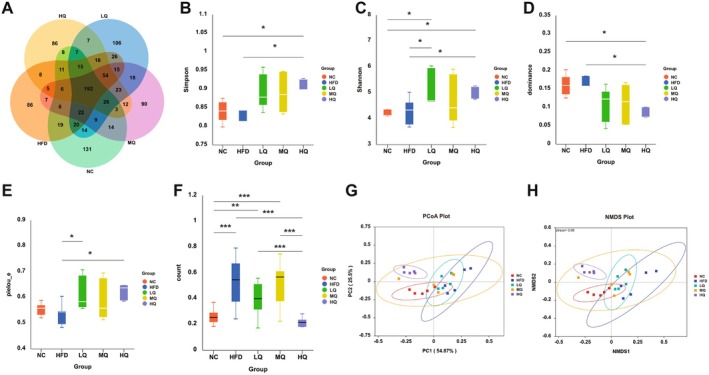
Richness, diversity, and evenness of the gut microbiota and principal coordinate analysis (PCoA). (A) Venn diagram showing the number of common and unique ASVs of gut microbiota. (B–E) Box plots showing the gut microbial α‐diversity based on Simpson, Shannon, dominance, and pielou_e indices. (F) Box plot showing the gut microbial β‐diversity. (G) Structural shifts (β‐diversity) presented by the weighted UniFrac PCoA based on the ASV abundance. (H) UniFrac distance‐based non‐metric multidimensional scaling (NMDS) analysis. Statistical significance is determined by *p* < 0.001 (***), *p* < 0.01 (**), and *p* < 0.05 (*), respectively.

Venn diagram analysis of ASVs revealed a total of 1062 ASVs across all samples, including 192 core ASVs shared among all experimental groups, with the normal control group (NC) displaying the highest number of unique ASVs (Figure 3A). Alpha‐diversity was quantified to evaluate microbial community richness, diversity, and evenness across the five experimental groups. The HFD group exhibited a modest, non‐significant increase in gut microbial richness, diversity, and evenness, compared to controls (Figure 3B–E and Figure S2). The Simpson index, Shannon index, dominance index, and pielou_e index of the HQ group were all greatly higher than those of the HFD group (Figure 3B–E). The results of the Bray–Curtis dissimilarity analysis indicated that the β‐diversity of the HQ group was markedly lower than that of the HFD group and the HQ group was closely related to the NC group (Figure 3F). The overall microbial structure results showed distinct clustering patterns among groups, with the HQ group exhibiting significant separation from the HFD group (Figure 3G). The results of NMDS analysis revealed significant separation between the HQ and HFD groups, with the HQ microbial profiles clustering more closely to the NC group (Figure 3H). Overall, these findings demonstrated that HQ supplementation attenuated HFD‐driven microbiota alterations.

### 3.3 Bacterial Taxa Associated With Quinoa Diet

**FIGURE 4:**
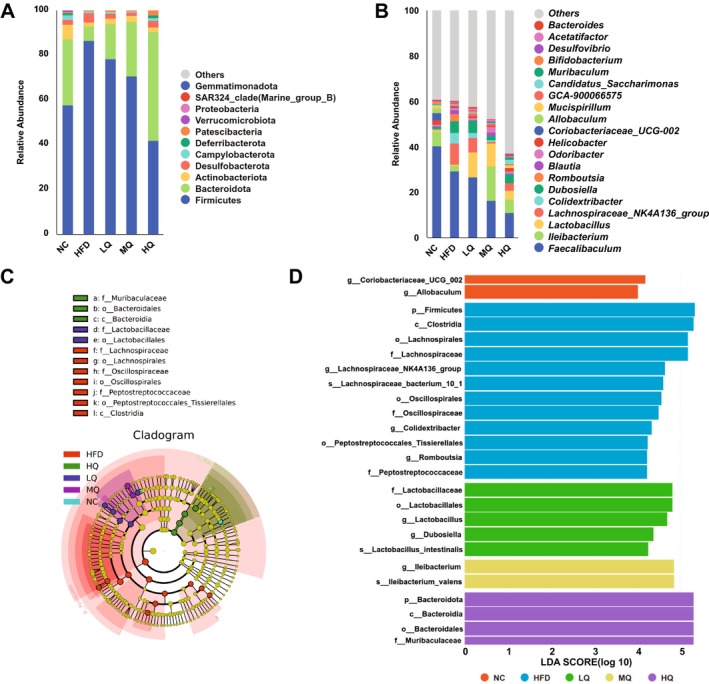
Taxonomic compositions of gut microbiota communities in different groups of mice, showing the relative abundances of gut microbiota at the (A) Phylum and (B) Genus levels, respectively. (C) Bacterial profiles in different groups of mice based on LEfSe analysis. (D) LDA scores derived from LEfSe analysis based on predicted microbial genes by PICRUSt, showing predictive functions of the microbial biomarkers. The taxonomic ranks include phylum (p__), class (c__), order (o__), family (f__), genus (g__), and species (s__).

The microbial taxa in the fecal ASV samples were annotated at the phylum and genus levels, respectively. At the phylum level (Figure 4A), the relatively high abundances were detected in Firmicutes, Bacteroidota, Actinobacteriota, and Desulfobacterota. The HFD group exhibited a significant increase in Firmicutes relative abundance compared to control mice, while decreased relative abundances were revealed in Bacteroidota and Actinobacteria (Figure 4A). HQ intervention reversed HFD‐driven Firmicutes enrichment but increased relative abundance in Bacteroidota. Notably, the elevated F/B ratio showed a positive correlation with obesity development (Magne et al. 2020). The HQ group exhibited a significantly reduced F/B ratio, suggesting that quinoa may alleviate obesity through gut microbiota modulation (Figure 4A). At the genus level, *Faecalibaculum*, *Ileibacterium*, *Lactobacillus*, and *Lachnospiraceae_NK4A136_group* were the relatively dominant bacterial taxa across all experimental groups (Figure 4B). The HFD group exhibited a significantly higher relative abundance of *Lachnospiraceae_NK4A136_group* compared to the NC group, while the relative abundance of *Lactobacillus* and *Ileibacterium* was significantly lower compared to controls (Figure 4B). The HQ group showed significantly reduced relative abundance in *Lachnospiraceae_NK4A136_group* but increased relative abundance in *Lactobacillus* and *Ileibacterium* compared to the HFD group (Figure 4B).

The LEfSe analysis was performed to identify marked differences in bacterial community predominance among different groups (Figure 4C,D). The HFD group exhibited a significant increase in Firmicutes relative abundance compared to the control group (Figure 4D and Table S3). The HQ group exhibited significantly higher relative abundances in Bacteroidota, Bacteroidia, Bacteroidales, and Muribaculaceae compared to other groups, with the highest LDA scores (> 5) detected for these taxa (Figure 4D and Table S3). Collectively, these results identified distinct microbial communities in HQ‐treated mice—including ubiquitous, low‐abundance, and HQ‐enriched taxa—that differed significantly from HFD‐induced obese mice.

### 3.4 Effects of Quinoa Diet on gWAT mRNA Expression of HFD Obese Mice

**FIGURE 5:**
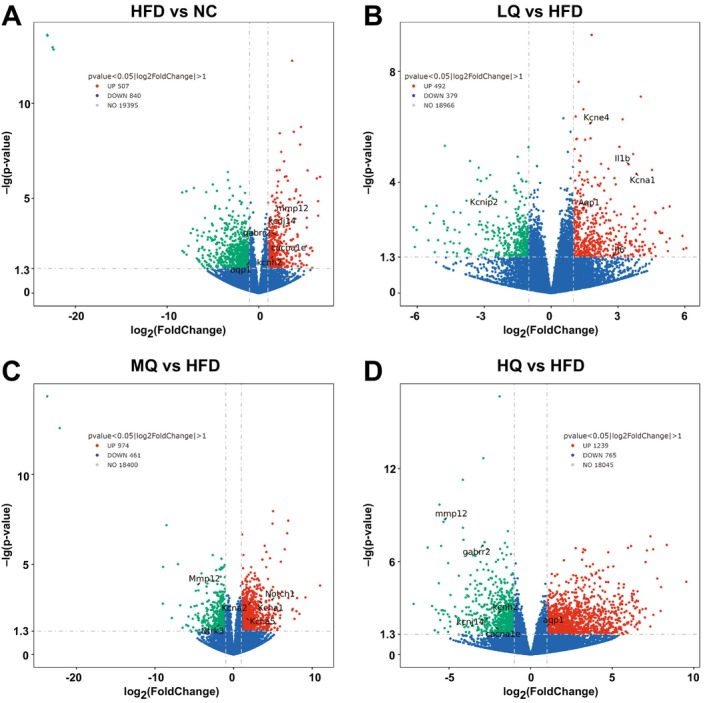
Identification of differentially expressed genes (DEGs) among different groups of mice based on volcano plots of comparative analysis of different groups of mice, i.e., the normal control (NC) group vs. HFD group (A), the HFD group vs. LQ group (B), the HFD group vs. MQ group (C), and the HFD group vs. HQ group (D). Significantly differential genes are colored in red (up‐regulated) and green (down‐regulated), respectively.

The global transcriptomics profiling was conducted among five groups of mice. Venn diagram analysis revealed a total of 14,852 DEGs across all samples, including 11,095 core DEGs shared among all experimental groups, with the NC group displaying the highest number of unique DEGs (Figure S3). Comparative transcriptomic analysis (based on the criteria of |log2 (Fold Change)| > 1.0 and *p* < 0.05) revealed significant dysregulation of gene expression in the HFD group relative to controls, with 507 significantly up‐regulated and 840 down‐regulated genes identified (Figure 5A). Compared to the HFD group, the LQ group showed 492 up‐regulated and 379 down‐regulated genes (Figure 5B), and the MQ group showed 974 up‐regulated and 461 down‐regulated genes (Figure 5C), with the largest numbers of DEGs detected in the HQ group (1239 up‐regulated and 765 down‐regulated) (Figure 5D). Together, these results implicated the pronounced regulatory effects of quinoa on mice with HFD.

### 3.5 Functional Enrichment Analysis of Differentially Expressed Genes

**FIGURE 6:**
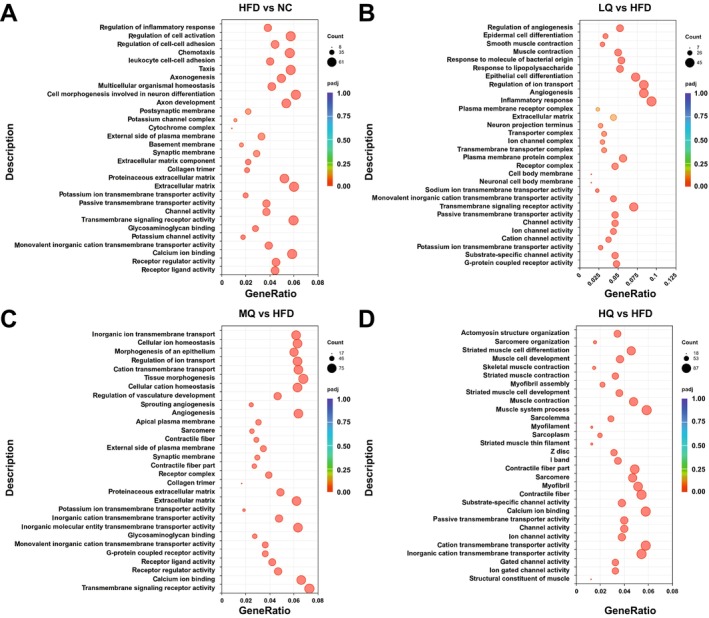
Scatter plots of the top 30 GO terms based on GO enrichment analyses of differentially expressed mRNAs identified in the pairwise comparisons of the four groups of mice, i.e., the normal control (NC) group vs. HFD group (A), the HFD group vs. LQ group (B), the HFD group vs. MQ group (C), and the HFD group vs. HQ group (D).

We further identified significantly enriched GO terms and KEGG pathways. Gene Ontology analysis identified significant enrichment of multiple metabolic pathways in the HFD group compared to the control group, including “passive transmembrane transporter activity,” “channel activity,” “calcium ion binding,” and “regulation of inflammatory response” (Figure 6A). In the LQ group, several signaling pathways were significantly enriched, including “channel activity,” “ion channel activity,” and “inflammatory response” (Figure 6B). In the MQ group, genes were greatly enriched in pathways such as “transmembrane signaling receptor activity,” “regulation of ion transport,” “calcium ion binding,” and “glycosaminoglycan binding” (Figure 6C). The HQ group showed significant enrichment in pathways including “passive transmembrane transporter activity,” “ion gated channel activity,” and “calcium ion binding” (Figure 6D). These results suggested that the administration of MQ and HQ reversed HFD‐triggered calcium ion binding, channel activity, and transmembrane transporter in gWAT.

**FIGURE 7:**
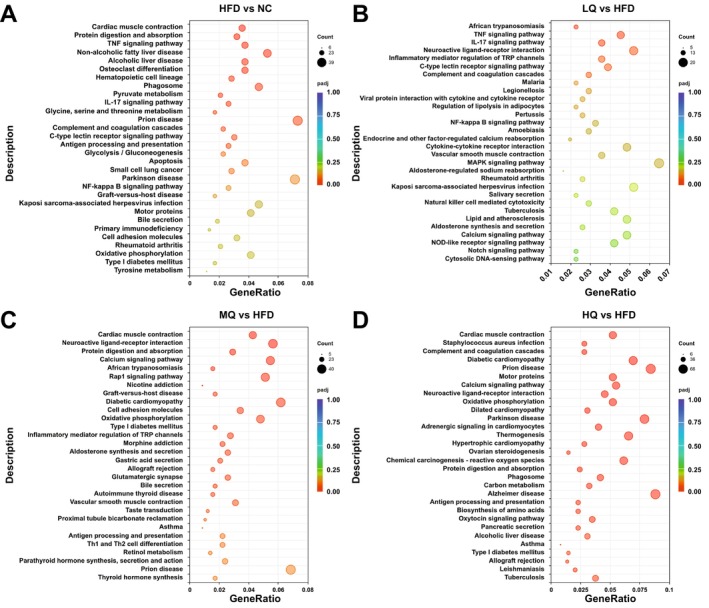
Scatter plots of the top 30 KEGG terms based on KEGG enrichment analyses of differentially expressed mRNAs identified in the pairwise comparisons of the four groups of mice, i.e., the normal control (NC) group vs. HFD group (A), the HFD group vs. LQ group (B), the HFD group vs. MQ group (C), and the HFD group vs. HQ group (D).

The results of KEGG enrichment analysis showed several pathways significantly enriched in the HFD group, including “cardiac muscle contraction,” “protein digestion and absorption,” “TNF signaling pathway,” and “non‐alcoholic fatty liver disease” (Figure 7A). In the LQ group, several signaling pathways were significantly enriched, including “African trypanosomiasis,” “TNF signaling pathway,” and “IL‐17 signaling pathway” (Figure 7B). In the MQ group, multiple signaling pathways exhibited significant enrichment, including “cardiac muscle contraction,” “protein digestion and absorption,” “calcium signaling pathway,” and “neuroactive ligand‐receptor interaction” (Figure 7C). Similarly, the HQ group showed significant enrichment in pathways including “cardiac muscle contraction,” “protein digestion and absorption,” “calcium signaling pathway,” and “neuroactive ligand‐receptor interaction” (Figure 7D). These results suggested that the administration of MQ and HQ reversed HFD‐triggered protein digestion and absorption and cardiac muscle contraction in gWAT.

### 3.6 Quinoa Treatment Protected Against HFD‐Enhanced Ion Channel‐Related Expression in gWAT

**FIGURE 8:**
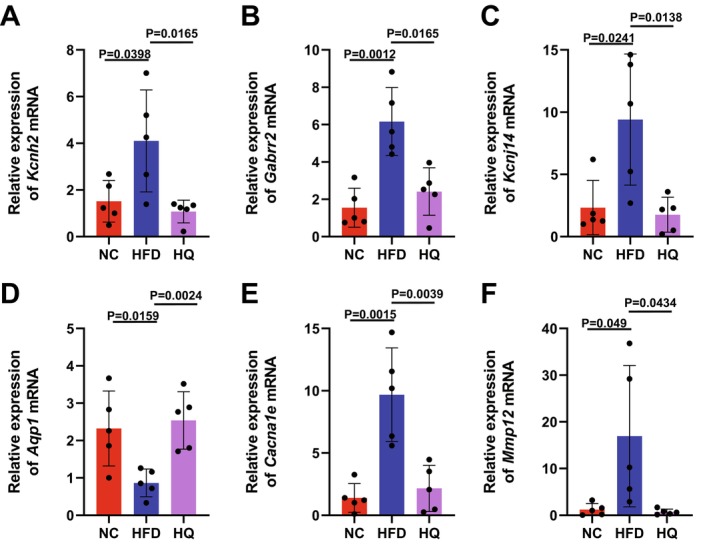
Differential gene expression analysis validated in the pairwise comparisons of the three groups of gWAT, i.e., the normal control (NC) group vs. HFD group and the HFD group vs. HQ group, showing the RT‐qPCR analysis of the relative mRNA expression of *Kcnh2* (A), *Gabrr2* (B), *Kcnj14* (C), *Aqp1* (D), *Cacna1e* (E), and *Mmp12* (F) in different groups of mice. *β‐Actin* transcript serves as an internal control for normalization. Data are analyzed using an unpaired Student's *t*‐test.

Our previous transcriptome results indicated that HQ supplementation reversed HFD‐induced alterations in “transmembrane transporter complex,” “ion gated channel activity,” and “calcium ion binding” in gWAT. To further validate the findings revealed by transcriptome analysis, RT‐qPCR analyses were performed based on NC, HFD, and HQ groups (Figure 8). The results validated the findings of transcriptome analysis, showing the higher expression levels in genes related to “transmembrane transporter complex” (Figure 8A,B), “ion gated channel activity” (Figure 8C,D), and “calcium ion binding” (Figure 8E,F) in HFD group than those in control group. Furthermore, HQ supplementation more effectively downregulated mRNA expression of genes involved in “transmembrane transporter complex”, “ion gated channel activity”, and “calcium ion binding” compared to HFD (Figure 8). Together, these results indicated that higher levels of ion channel‐related genes were significantly associated with the incidence of HFD‐induced obesity, and HQ ameliorated HFD‐induced alterations in the expression of genes associated with ion channel in gWAT.

The maintenance of lipid homeostasis in gWAT plays a critical regulatory role in obesity development (Axelrod et al. 2023; Chen et al. 2020; Sakers et al. 2022). Our transcriptome analysis revealed that HQ supplementation greatly antagonizes HFD‐induced lipid metabolism dysfunction in gWAT (Tables S4 and S5). To further validate the findings from the transcriptome analysis, RT‐qPCR was conducted on the NC, HFD, and HQ groups. The RT‐qPCR results demonstrated downregulating mRNA expression of key metabolic genes involved in lipid metabolism in the HQ group compared to HFD diets, including *Apoc4*, *Lbp*, *Apln*, *Lep*, *Scd2*, and *Ffar2* (Figure S4A–F and Tables S4 and S5). Therefore, these results indicated that HQ could alleviate HFD‐induced obesity by inhibiting lipid metabolism in gWAT.

## 4 Discussion

Obesity has emerged as a growing global health epidemic, recognized both as a preventable risk factor for non‐communicable disorders and as a distinct disease entity. Quinoa has emerged as a global superfood due to its exceptional nutritional composition and unparalleled abiotic stress resistance, including resistance to drought, frost, and salinity (Hlásná Cepková et al. 2022). In this study, an obesity model was established in female C57BL/6J mice through 8‐week HFD feeding, and the mice were concurrently fed a HFD with varying dosages of quinoa. The anti‐obesity mechanisms investigated included: (1) changes in weight and adiposity; (2) gut microbial shifts; and (3) differential gene expression patterns.

### 4.1 Quinoa Potentially Modulates the Ca^2+^ and Sensory Neuronal Signaling Through Inhibition of Ion Channels

Adipose tissue constitutes the principal depot for triglyceride storage and fatty acid mobilization, supplying energy substrates to peripheral organs. It is also integral to the regulation of appetite, glucose homeostasis, insulin sensitivity, and thermoregulation (Rosen and Spiegelman 2014). This regulatory capacity is facilitated by a sophisticated bidirectional innervation of sympathetic and sensory nerves, forming a critical feedback loop between adipose depots and the central nervous system (Wang and Ye 2023). While exogenous compounds that activate adipose sensory neurons have been identified, their endogenous counterparts have yet to be identified. Accumulating evidence reveals that sensory neuronal pathways, particularly through Runx3 and parvalbumin, play pivotal roles in coordinating whole‐body metabolic balance, lipid accumulation, and glycemic control (Passini et al. 2025). As a key secondary messenger, cytosolic Ca^2+^ regulates adipocyte metabolism and differentiation by modulating adipogenic gene expression (Zhai et al. 2020). Intracellular Ca^2+^ regulates adipocyte metabolism and differentiation through secondary messenger functions (Zhai et al. 2020), and KCNH2 deficiency increases calcium ion influx and elevates intracellular calcium ion levels (Yuan et al. 2024). In our study, the MQ group exhibited restored expression of HFD‐dysregulated genes (*Runx3* and *parvalbumin*) while concurrently counteracting HFD‐induced obesity (Figure 1A and Table S4). The HQ group demonstrated significantly increased *KcnH2* gene expression while effectively attenuating HFD‐induced obesity. These findings suggest that both MQ and HQ exert anti‐obesity effects against HFD‐induced adiposity via distinct mechanisms, i.e., MQ exerts its anti‐obesity effects primarily via modulation of adipose tissue sympathetic innervation, while HQ predominantly through calcium signaling pathways. While quinoa demonstrates regulatory effects on the gene expression of adipose sensory neurons and calcium‐associated factors, the functional consequences on neuronal signaling and calcium dynamics need further verification. Meanwhile, the potential regulatory effects of quinoa as an activator of adipose sensory neurons upon the influx of calcium ions into adipocytes need further investigation.

### 4.2 Modulatory Effects of Quinoa Bioactives on Diet‐Induced Obesity and Metabolic Dysregulation

Quinoa possesses exceptional nutritional value characterized by its rich composition of proteins, lipids, dietary fibers, essential vitamins and minerals, along with an optimal balance of essential amino acids (Abugoch James 2009). Furthermore, quinoa contains diverse bioactive metabolites, including saponins, phenolic acids, flavonoids, terpenoids, and phytosterols, etc. (Ren et al. 2023; Sharma et al. 2025). These bioactive metabolites demonstrate multiple therapeutic properties beneficial for human health (Estrada et al. 1998; Graf et al. 2014; Hu et al. 2017; Yao et al. 2014). While quinoa is recognized as a functional food with demonstrated anti‐obesity properties, the precise mechanisms underlying its potent effects remain to be fully elucidated. Previous research reported that quinoa saponins significantly suppressed adipocyte differentiation through downregulation of key adipogenic transcription factors (Teng et al. 2020; Yao et al. 2015), and quinoa saponins greatly ameliorated inflammation, obesity, and metabolic disorders by modulating gut microbiota (Li, Song, et al. 2022). Notably, food intake monitoring indicated a marked suppression in the quinoa group (Figure 1C), potentially attributed to appetite‐suppressing saponins present in quinoa. The previous investigation evaluated the anti‐obesity potential of 20‐hydroxyecdysone (20E)‐fortified quinoa extract, focusing on both weight gain prevention and adipose tissue adipokine modulation (Foucault et al. 2012). Consequently, it is proposed that the mechanisms by which HQ supplementation suppresses obesity and downregulates adipokine expression are potentially dependent on saponins and 20E (Figure 1A and Figure S4). Therefore, the collective action of diverse bioactive compounds in quinoa warrants deeper mechanistic elucidation.

### 4.3 Quinoa Inhibits gWAT Adipogenesis Through Modulation of Lipid Metabolism

Obesity represents a major public health burden, driving metabolic dysfunction and lipid metabolism disorders. Emerging evidence suggests that quinoa may serve as an effective nutritional strategy for improving glycemic regulation and metabolic dysfunction (Ren et al. 2023). Aquaporins (AQPs) have emerged as significant contributors to obesity pathogenesis through their roles in lipid accumulation. Targeted modulation of AQP1 expression provides a therapeutic approach for obesity management and fat deposition control (da Silva and Soveral 2017). Moreover, the adipokine apelin signals through the APJ receptor and is associated with metabolic disorders including obesity and diabetes (Li, Cheng, et al. 2022). Statin‐mediated LDL‐C reduction is associated with a concomitant increase in serum apelin levels in dyslipidemic patients (Tasci et al. 2009). Our findings suggest that HQ regulates lipid metabolism potentially through regulating ion channel (AQP1) and adipokine expression (apelin) (Figures 8D and Figure S4C). These data suggest quinoa may regulate lipid metabolism through two separate pathways: indirect regulation via ion channel gene expression and direct regulation through lipid metabolism gene modulation. Nevertheless, it remains a subject for future investigation to determine if quinoa modulates lipid metabolism gene expression through its effects on ion channel‐associated genes in WAT. Dietary fiber (DF), the 7th essential nutrient, encompasses indigestible polysaccharides (cellulose, hemicellulose, etc.) that modulate gut microbiome and host metabolic homeostasis, offering protection against HFD‐induced obesity and metabolic syndrome (Ren et al. 2023; Wang et al. 2021). Transcriptomic analysis further revealed that DF upregulated the expression levels of key lipid metabolism‐related genes (Wang et al. 2021). Therefore, in addition to bioactive compounds, quinoa fiber may ameliorate HFD‐induced obesity by modulating gut microbiota and lipid metabolism. Beyond quinoa extract and polysaccharides, quinoa fiber and protein harbor numerous additional bioactive compounds, and several of these compounds may contribute to its anti‐obesity effects and warrant further investigation.

### 4.4 Quinoa Consumption May Confer Obesity Resistance Through Modulation of Adipose Homeostasis

Previous studies have demonstrated that supplementation of immunonutritionally active fraction (quinoa glucosides and immunonutritional protease inhibitors) from quinoa potently induces innate immune responses, which are critical for effectively maintaining macrophage polarization in HFD‐induced murine models (Laparra and Haros 2019; Srdić et al. 2020). Macrophage polarization states exert profound effects on adipose tissue homeostasis and are centrally involved in obesity induction (Makassy et al. 2025). Notably, quinoa seeds comprise bioactive compounds that effectively suppress the overproduction of inflammatory markers, underscoring their anti‐inflammatory potential. Our study demonstrates that HFD modulates the activation of immune cell differentiation processes within gWAT (Figure 7A). Whereas, HQ significantly regulated the expression of genes related to antigen processing and presentation in gWAT (Figure 7D), suggesting that HQ may inhibit HFD‐induced adipose tissue homeostasis disruption through distinct immunomodulatory mechanisms. However, the functional impact of these two pathways on macrophage polarization and the immune microenvironment needs further exploration. Leptin, produced and secreted by WAT into circulation, with the induced PD‐1 subsequently suppressing tumor‐associated macrophage metabolic activity (Bader et al. 2024). HQ effectively regulates antigen processing and presentation‐related genes and leptin expression (Figure 7D and Figure S4D); therefore, HQ may counteract HFD‐induced alterations in adipose tissue immune cell activity through modulation of adipose tissue homeostasis. However, further studies are needed to determine if quinoa attenuates HFD‐induced obesity by reducing macrophage infiltration and modulating inflammatory mediators in adipose tissue.

### 4.5 Impact of Quinoa Consumption on Gut Microbiota Modulation

Obesity is significantly correlated with phylum‐level remodeling in gut microbiota composition, and functional changes in microbial genes and metabolic pathways (Turnbaugh et al. 2009). Accumulating evidence indicates that obese subjects, consistently demonstrate an increased F/B ratio relative to normal‐weight individuals, positioning this metric as a candidate biomarker for metabolic dysregulation. For example, an enhanced F/B ratio is widely characterized as a microbial signature of obesity in human and animal studies (Magne et al. 2020). Moreover, gut microbiome profiling revealed that lower relative abundance of Bacteroidota, Bacteroidia, Muribaculaceae, and Bacteroidales indicates their potential involvement in obesity progression (Li et al. 2023). In the present study, quinoa supplementation demonstrated a dose‐dependent anti‐obesity effect (Figure 1A). Specifically, quantitative analyses revealed that quinoa exhibited dose‐gradient alterations in the F/B ratio along with enriched abundances of Muribaculaceae, Bacteroides, Bacteroidota, and Bacteroidaceae, indicating that dose‐dependent microbial remodeling represents a potential anti‐obesity mechanism. The precise mechanisms through which quinoa exerts its dose‐dependent effects on microbial abundance warrant future exploration.

### 4.6 The Regulatory Role of Quinoa on WAT in Female Obese Mice

Recent epidemiological data indicate that the prevalence of obesity among females now substantially exceeds that observed in males (Broughton and Moley 2017); “Health Effects of Overweight and Obesity in 195 Countries over 25 Years,” (GBD 2015 Obesity Collaborators 2017). This trend is of particular clinical concern due to the strong association between obesity in women and a spectrum of serious comorbidities (Broughton and Moley 2017; Cheshmeh et al. 2022). It is well established that women typically have a higher total adiposity than men, yet are characterized by a more favorable distribution of fat, with a greater amount of metabolically protective adipose tissue. The postmenopausal period, marked by a decline in circulating estrogen, is associated with a shift in fat distribution towards visceral depots, a loss of protective fat, and the development of impaired insulin signaling (Ayesh et al. 2025; Lee and Fried 2017; Steiner and Berry 2022). Consequently, elucidating the molecular mechanisms of visceral fat accumulation in females and establishing effective strategies to combat obesity in women are urgently required. Although dietary supplementation with quinoa has previously been shown to elicit anti‐obesity effects in male mouse models, its potential efficacy in females has not been investigated (An et al. 2021; Félix‐Soriano et al. 2021; Qiao et al. 2018; Rodrigues e Lacerda et al. 2025). Here, we report that quinoa administration produces analogous beneficial effects in female mice, significantly reducing body weight gain, visceral fat accumulation and decreasing the Firmicutes‐to‐Bacteroidetes (F/B) ratio (Figures 1A,E and 4A). While female mice exhibit resistance to obesity via estrogen‐mediated mechanisms (Ayesh et al. 2025; Lee and Fried 2017; Steiner and Berry 2022), estrogen does not appear to compromise the anti‐obesity efficacy of quinoa under HFD conditions (Figure 1A,E). The specific role of estrogen in the quinoa‐mediated regulation of metabolic health warrants further investigation. Previous studies on quinoa have largely centered on its effects on hepatic oxidative stress (Song et al. 2021), gut‐brain axis signaling (Wang et al. 2022), type 2 diabetes modulation (Zheng et al. 2023), and gut microbiota composition in the context of obesity (Liu et al. 2018). However, its influence on WAT remodeling and function throughout this process has not been directly examined. WAT is a dynamic endocrine organ that orchestrates complex metabolic processes in physiological states (Schoettl et al. 2018). Its pathological expansion is linked to a spectrum of adverse phenotypic alterations, such as chronic inflammation, fibrotic remodeling, localized hypoxia, aberrant adipokine production, and impaired mitochondrial bioenergetics (Kusminski et al. 2016). Here, we present the first profiling of quinoa‐induced gene transcriptional changes in WAT within the context of obesity. Our results demonstrate that HQ supplementation substantially reverses HFD‐induced gene dysregulation in WAT (Figure 8). As quinoa is digested and metabolized within the gastrointestinal tract, we hypothesize that its mechanism of action may involve direct modulation of gut microbial communities. In parallel with these microbial shifts, we detected corresponding alterations in WAT gene expression profiles. Taken together, these observations lead us to propose that the transcriptional remodeling in WAT is potentially mediated through quinoa‐driven restructuring of the gut microbiota. Nevertheless, the causal relationship among quinoa intake, microbial ecology, and adipose tissue gene expression remains to be rigorously established through further experimentation.

## 5 Conclusion

In conclusion, based on a comprehensive approach with validations, our study explored gut microbial composition and proposed Bacteroidota, Bacteroidia, Bacteroidales, and Muribaculaceae as novel biomarkers for anti‐obesity function in HQ supplementation. Moreover, our findings demonstrated that HQ administration restructured gWAT dysfunction by modulating ion channel activity in gWAT. This work provides novel insights and guidance for developing new strategies to combat diet‐induced obesity, as well as potential evidence for the weight‐control effects of dietary supplements.

## Funding

This research was supported by the Department of Science and Technology of Jilin Province, China (20210101220JC) and the Department of Education of Jilin Province, China (JJKH20240231KJ).

## Ethics Statement

All animal procedures were performed in accordance with the Institutional Animal Care and Use Committee (IACUC) guidelines under an approved protocol by the IACUC of Jilin Engineering Normal University (Approval number 2024SY10502). The experimental procedures conducted in this study complied with approved guidelines.

## Conflicts of Interest

The authors declare no conflicts of interest.

## Supporting information


**Figure S1:** Morphological features of small intestine tissue in the five groups of mice, showing (A) H&E staining (scale bar = 100 μm) and (B) Average length of villi in small intestine based on H&E staining. Statistical differences are based on *p* < 0.001 (***) and *p* < 0.05 (*), respectively; ns, not significant.
**Figure S2:** Box plots showing the gut microbial α‐diversity based on Chao1 index.
**Figure S3:** Venn diagram showing the number of common differentially expressed mRNAs of the different groups of mice.
**Figure S4:** Differential gene expression analysis validated in the pairwise comparisons of the three groups of gWAT, i.e., the NC group vs. HFD group and the HFD group vs. HQ group, showing the RT‐qPCR analysis of the relative mRNA expression of *Apoc4* (A), *Lbp* (B), *Apln* (C), *Lep* (D), *Scd2* (E), and *Ffar2* (F) in different groups of mice. *Actin* transcript serves as an internal control for normalization. Data are analyzed using unpaired Student's *t*‐test.

**Table S1:** Nutrient composition of feed.

**Table S2:** RT‐qPCR primer sequence.

**Table S3:** LEfSe analysis of the characteristic gut microbiota.

**Table S4:** Compare the differentially expressed genes between the NC and HFD groups.

**Table S5:** Compare the differentially expressed genes between the HFD and HQ groups.

## Data Availability

Raw data have been deposited in the NCBI Sequence Read Archive (SRA) database and are now available under accession number PRJNA1279820. Additional information and requests for additional data and code should be directed to Zhandong Li (lizd591@jlenu.edu.cn).
