# Supplementary material for: Quinoa Ameliorates High‐Fat Diet‐Induced Obesity in Female Mice by Regulating Gut Microbiota and Adipogenesis
Source: Food Sci Nutr. 2025 Nov 25;13(12):e71250. doi: 10.1002/fsn3.71250 (PMC12645152; doi:10.1002/fsn3.71250)
Supplement: Supplementary file 1 — Figure S1: Morphological features of small intestine tissue in the five groups of mice, showing (A) H&E staining (scale bar = 100 μm) and (B) Average length of villi in small intestine based on H&E staining. Statistical differences are based on p < 0.001 (***) and p < 0.05 (*), respectively; ns, not significant. Figure S2: Box plots showing the gut microbial α‐diversity based on Chao1 index. Figure S3: Venn diagram showing the number of common differentially expressed mRNAs of the different groups of mice. Figure S4: Differential gene expression analysis validated in the pairwise comparisons of the three groups of gWAT, i.e., the NC group vs. HFD group and the HFD group vs. HQ group, showing the RT‐qPCR analysis of the relative mRNA expression of Apoc4 (A), Lbp (B), Apln (C), Lep (D), Scd2 (E), and Ffar2 (F) in different groups of mice. Actin transcript serves as an internal control for normalization. Data are analyzed using unpaired Student's t‐test. [file FSN3-13-e71250-s003.docx]

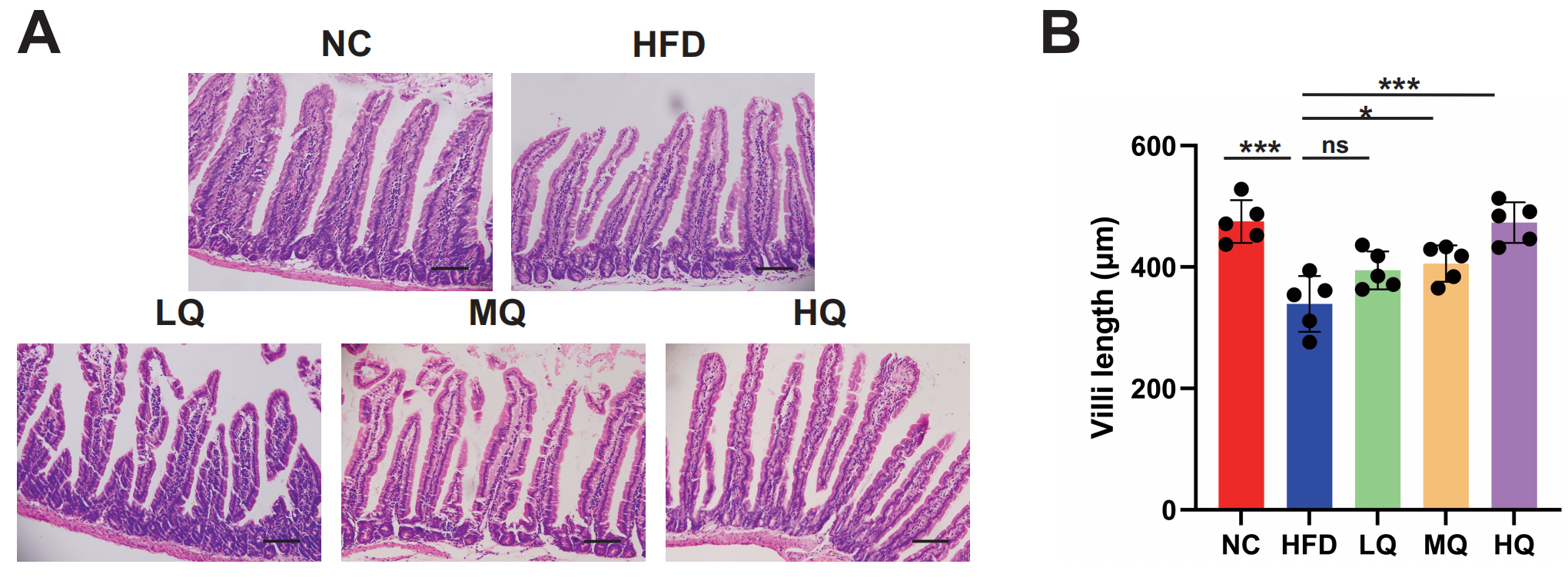


**Supplementary Figure S1.** Morphological features of small intestine tissue in the five groups of mice, showing (A) H&E staining (scale bar = 100 μm) and (B) Average length of villi in small intestine based on H&E staining. Statistical differences are based on *P* < 0.001 (***) and *P* < 0.05 (*), respectively; ns, not significant.


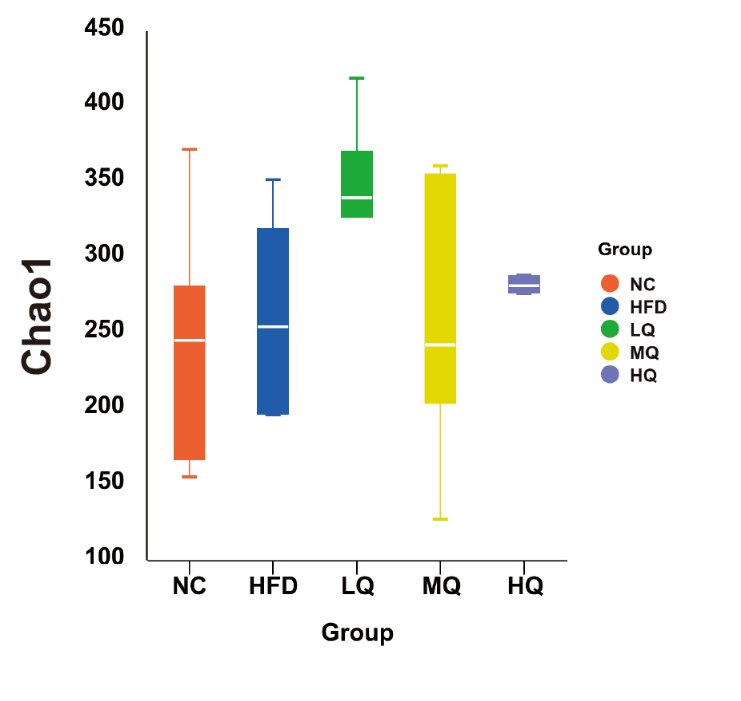


**Supplementary Figure S2.** Box plots showing the gut microbial α-diversity based on Chao1 index.


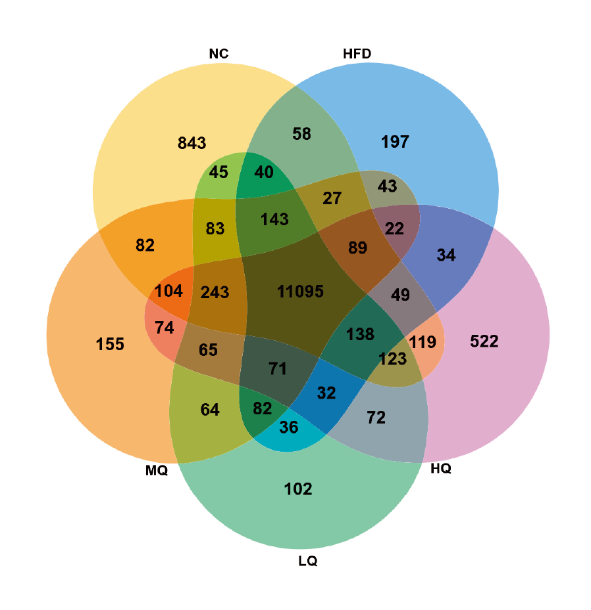


**Supplementary Figure S3.** Venn diagram showing the number of common differentially expressed mRNAs of the different groups of mice.


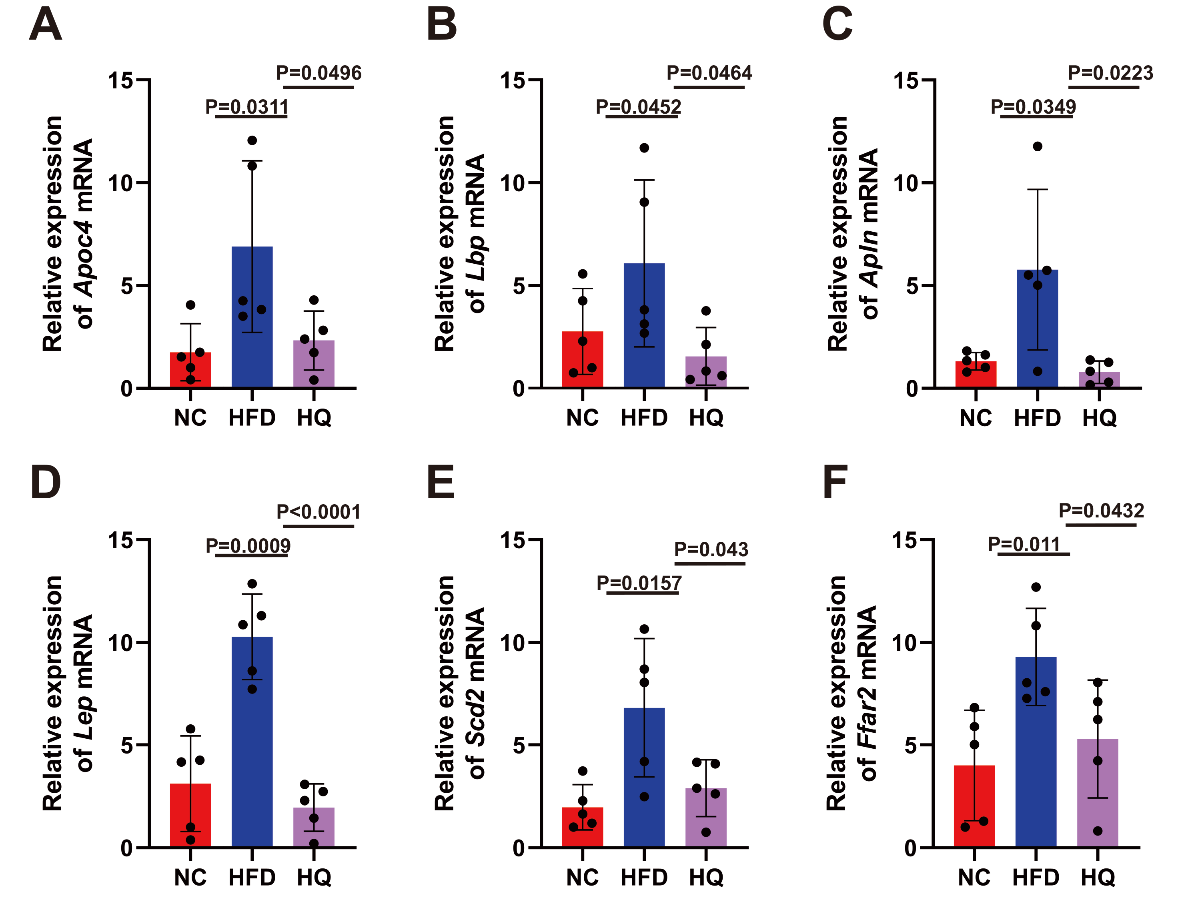


**Supplementary Figure S4.** Differential gene expression analysis validated in the pairwise comparisons of the three groups of gWAT, i.e., the NC group vs. HFD group and the HFD group vs. HQ group, showing the RT-qPCR analysis of the relative mRNA expression of *Apoc4* (A), *Lbp* (B), *Apln* (C), *Lep* (D), *Scd2* (E), and *Ffar2* (F) in different groups of mice. *Actin* transcript serves as an internal control for normalization. Data are analyzed using unpaired Student’s t-test.
